# Sensorineural hearing loss in a case of congenital Zika virus^☆^

**DOI:** 10.1016/j.bjorl.2016.06.001

**Published:** 2016-06-30

**Authors:** Mariana de Carvalho Leal, Lilian Ferreira Muniz, Silvio da Silva Caldas Neto, Vanessa van der Linden, Regina Coeli Ferreira Ramos

**Affiliations:** a Universidade Federal de Pernambuco (UFPE), Departamento de Cirurgia, Serviço de Otorrinolaringologia, Recife, PE, Brazil; b Universidade Federal de Pernambuco (UFPE), Departamento de Fonoaudiologia, Recife, PE, Brazil; c Associação de Assistência à Criança Deficiente de Pernambuco, Clínica de Neurologia Infantil, Recife, PE, Brazil; d Universidade Federal de Pernambuco (UFPE), Divisão de Doenças Infecciosas, Departamento de Clínica Mèc)dica, Recife, PE, Brazil

## Introduction

Zika virus (ZIKV) was first isolated in 1947 and, in 1952, antibodies against it were identified in humans. It is a flavivirus that causes a dengue-like syndrome, including fever, headache, malaise, arthralgia, myalgia, maculopapular rashes, and conjunctivitis. After a long period of sporadic reports of human infections, two major outbreaks occurred in 2007, in Micronesia, and in 2013•2014, in French Polynesia, from where it has spread to other countries, including Brazil, particularly in the northeast,^1^ in 2015.

## Case description

A newborn from a twin pregnancy was delivered by caesarian section during the 37th week of gestation. On physical examination the only apparent anomalies were microcephaly (cephalic perimeter at birth = 28 cm) and bilateral clubfoot. The other twin was normal. His mother, who lived in the state of Pernambuco, in northeastern Brazil, reported a rash and fever on about the 28th day of pregnancy.

**Figure 1:**
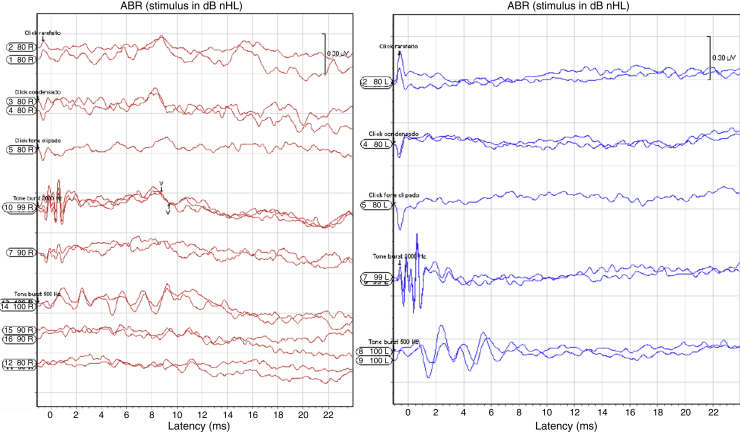
Auditory brainstem evoked responses. Note the bilateral profound deafness.

Transient otoacoustic emissions were absent. The auditory brainstem response (ABR) to clicks was then measured and repeated one month later. On both occasions, no response was obtained from the left ear. In the right ear, there was a response to clicks at 99 dB (Fig. 1). Frequency-specific ABR with tone bursts was done, confirming bilateral profound hearing loss. The ear, nose and throat examination was otherwise normal. Behavioral auditory evaluation was done using instruments of known frequency range at 60 cm from the pinna. There was no response even for high intensity stimulus. The cochleopalpebral reflex was absent and there was no response to voice stimulus at 100 dB.

**Figure 2:**
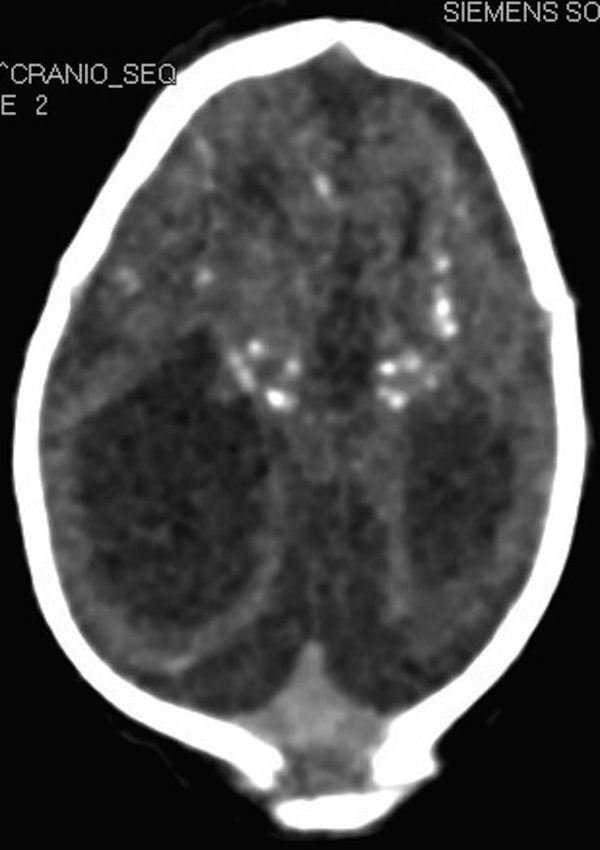
Axial encephalic tomography. Note the malformation of cortical development and calcifications.

Computerized tomography revealed diffuse bilateral reduction of cerebral parenchyma, ventriculomegaly, malformation of cortical development, with simplified gyral pattern and multiple cerebral calcifications predominantly in the basal ganglia and cortical-subcortical regions (Fig. 2). Capture ELISA was positive for ZIKV IgM on the cerebrospinal fluid (CSF). Polymerase chain reaction (PCR) tests for *Herpes simplex* in serum were negative; PCR tests for cytomegalovirus in cerebrospinal fluid, serum and urine were also negative; toxoplasmosis IgM was negative and IgG positive in the mother's blood, and IgM negative and IgG positive in the newborn's blood but at lower levels than in the mother. Other causes of microcephaly, such as alcoholism or genetic disorders, were also excluded.

On ophthalmologic evaluation, cornea and lens were found normal. There was high level of myopia, the fundus presented bilateral macular coloboma; there was a large area of exposed sclera at right and optic nerve hypoplasia at left. Glaucoma was noted bilaterally. The twin brother was completely normal both on clinical examination and blood and CSF tests.

At two months the child presented with major irritability. An electroencephalogram showed focal activity. Unstable cervical control, poor interaction, no evidence of visual contact and global retardation of development were noted. He was operated during the third month to correct the clubfeet, and on the fourth month for glaucoma. A neuromotor stimulation program was initiated.

## Discussion

In 2015, the Brazilian Ministry of Health recorded a 15-fold increase in the number of cases of microcephaly in the state of Pernambuco, and in November 28th 2015 it confirmed a correlation between gestational Zika infection and microcephaly in newborn infants.^2^ Since then intensive measures have been taken to control the viral outbreak and a task force has been created to investigate other possible deleterious effect of ZIKV intrauterine infection.

We believe that this is the first report of hearing loss associated with gestational Zika infection in the world literature. We must stress that all the other infectious causes of congenital hearing loss were excluded by serologic tests, that the rash and fever suffered by the mother occurred during an outbreak of ZIKV on that same region, and that IgM for ZIKV was detected in CSF. It is also of note that his twin brother did not have signs of ZIKV infection and was absolutely healthy. This highlights the potential for this viral disease to cause disastrous consequences for the fetus when acquired during pregnancy. The pathological mechanisms involved in causing this functional damage is far from being elucidated, but it is probable that they follow the routes common to other congenital viral disorders such as cytomegalovirus or rubella.

Microcephaly has a variety of causes which include congenital infections such as cytomegalovirus, herpes simplex type II, human immunodeficiency virus, rubella and varicella zoster virus.^3^ The degree of pathology can vary from slight cranial malformation with normal neurological development to severe, with variable degrees of mental retardation. The abnormalities seen by tomography, as well as the clinical findings, point to a major neurologic impairment in this case.

These central lesions can be the origin of the auditory impairment of this child, but the absence of otoacoustic emissions indicates that a peripheral (cochlear) damage is more likely to be the cause. Patients with rubella and cytomegalovirus can also present with microcephaly, but it is well established that in these cases the auditory malfunction is secondary to a peripheral problem^4^.

It is still uncertain if the tissue damages caused by the intrauterine ZIKV infection are an expression of a direct effect of the virus itself or of an immune reaction from the host. Further histologic studies are necessary to determine the exact pathogenesis of the disease.

## Conclusion

This case indicates that the occurrence of ZIKV must be carefully monitored for during prenatal visits in affected regions and must be considered as a possible cause of congenital deafness when a history of a rash and fever occurs during the gestational period, especially during the first trimester. In hearing assessment protocols for neonates, mother's infection by ZIKV should be included among the risk factors for hearing loss.

## Conflicts of interest

The authors declare no conflicts of interest.
